# Effects of Tetracycline Susceptibility and Virulence in an *acrR* Mutant of *Erwinia amylovora*

**DOI:** 10.4014/jmb.2608.08022

**Published:** 2026-10-07

**Authors:** Jeong Ho Choi, Yeon Ju Kim, Hyun Ju Choi, Duck Hwan Park

**Affiliations:** 1 Interdisciplinary Program in Smart Agriculture, Kangwon National University, Chuncheon 24341, Republic of Korea; 2 Plant Medicine Program, Division of Bioresource Sciences, College of Agriculture and Life Sciences, Kangwon National University, Chuncheon 24341, Republic of Korea

**Keywords:** *Erwinia amylovora*, Tetracycline susceptibility, Multidrug efflux, *acrR*, lipopolysaccharide, Virulence

## Abstract

This study investigated whether AcrR-mediated regulation of the *acrAB* efflux system is associated with tetracycline susceptibility in *Erwinia amylovora*. We compared tetracycline susceptibility, *acrAB* expression, intracellular tetracycline accumulation, intracellular pH, ATP-associated luminescence, envelope-associated phenotypes, virulence, and responses to exogenous acyl-homoserine lactones (AHLs) among the wild type (WT), an *acrR* mutant (Δ*acrR*), the same mutant after prior tetracycline exposure (Δ*acrR*^Tc^), and an *acrR*-complemented mutant (Δ*acrR*(*acrR*)). Relative to WT, Δ*acrR* showed fourfold increases in tetracycline MIC and MBC, whereas Δ*acrR*^Tc^ showed 16- and 32-fold increases, respectively. Δ*acrR*^Tc^ also showed markedly increased *acrA* and *acrB* expression, reduced intracellular tetracycline accumulation, lower intracellular pH, and reduced ATP-associated luminescence, suggesting that prior tetracycline exposure was associated with an enhanced AcrAB response in the *acrR*-deficient background. Δ*acrR*^Tc^ further displayed altered cell-envelope ultrastructure, increased *waaL* expression and lipopolysaccharide (LPS)-associated band intensity, reduced biofilm formation, and a weaker cellulose-associated colony phenotype, whereas amylovoran production and levansucrase-associated activity remained unchanged. In the pathogenicity assay, Δ*acrR*^Tc^ produced weaker necrotic symptoms, lower bacterial populations, and reduced *in planta* *dspE* expression. Among the AHLs tested, OHHL produced a distinct virulence pattern, with Δ*acrR* showing reduced disease severity relative to WT and Δ*acrR*(*acrR*), whereas this separation was not observed with HHL or OOHL; Δ*acrR*^Tc^ remained less virulent under all AHL conditions. These findings support tetracycline as an AcrAB substrate and suggest that prior tetracycline exposure can strengthen the subsequent AcrAB-associated response. The OHHL-specific phenotype further raises the possibility that OHHL may also serve as a substrate of the AcrAB efflux system.

## Introduction

Fire blight, caused by the Gram-negative bacterium *Erwinia amylovora*, is a serious disease of apple and pear. Since the first confirmed outbreaks in the Republic of Korea in 2015, the disease has spread to major apple- and pear-growing regions, leading to continued disease monitoring, removal of infected trees, and preventive bactericide application during bloom [1-3]. Antibiotics are still used to control fire blight in regions where their use in agriculture is permitted [4, 5]. However, repeated antibiotic exposure can select for bacterial populations with reduced susceptibility. High-level resistance to oxytetracycline and streptomycin mediated by an IncX plasmid has been reported in *E. amylovora* isolates collected from commercial pear orchards in California [6]. This finding highlights the need to understand both acquired resistance genes and chromosomal mechanisms that affect tetracycline susceptibility in *E. amylovora*.

In Gram-negative bacteria, tetracycline susceptibility is influenced by both acquired *tet* resistance genes and intrinsic multidrug efflux systems. AcrAB–TolC is a major resistance–nodulation–division (RND)-type efflux system composed of the inner-membrane transporter AcrB, the periplasmic adaptor AcrA, and the outer-membrane channel TolC [7]. The *acrAB* operon is locally repressed by AcrR [8]. In *Escherichia coli*, loss of AcrAB increases susceptibility to tetracycline and several other antibiotics [9]. In *Enterobacter aerogenes*, a frameshift mutation in *acrR* was associated with multidrug resistance, whereas introduction of wild-type *acrR* reduced the resistance phenotype [10]. Similarly, in *Agrobacterium tumefaciens*, AcrR represses the *acrABR* operon, and AcrAB contributes to resistance to tetracycline and several other toxic compounds [11]. Previous studies in *E. coli* have further shown that stepwise or prolonged tetracycline exposure can increase the expression of proton gradient-dependent multidrug efflux systems and accompany progressive reductions in tetracycline susceptibility [12, 13]. In particular, *acrAB*-*tolC* expression can become more pronounced during continued tetracycline exposure, indicating that the magnitude of the efflux-associated response may depend not only on the regulatory background but also on the extent of antibiotic exposure [12, 13]. Together, these findings show that AcrR-mediated regulation and AcrAB function influence antibiotic susceptibility and provide a rationale for examining whether prior tetracycline exposure modifies the phenotype associated with *acrR* disruption.

The AcrAB–TolC system is also present and functional in *E. amylovora*, although previous studies have focused mainly on its roles in phytoalexin resistance, host colonization, and virulence. Disruption of *acrB* or *tolC* increases susceptibility to plant-derived phytoalexins and several antimicrobial compounds, including tetracycline, while reducing bacterial survival and virulence in apple plants [14, 15]. More recently, stepwise oxytetracycline exposure of *E. amylovora* was reported to be associated with the emergence of mutations in AcrR during the acquisition of increased tetracycline resistance [16]. Thus, although previous studies have suggested an association of the AcrR–AcrAB system with tetracycline susceptibility in *E. amylovora*, the AcrR-dependent regulatory and physiological response to tetracycline has not been directly characterized. In particular, it remains unclear how AcrR-dependent regulation of *acrAB* is related to intracellular tetracycline accumulation and whether prior tetracycline exposure is accompanied by coordinated changes in cellular physiology, cell-envelope phenotypes, and virulence in an *acrR*-deficient background.

Based on this background, we compared the effects of *acrR* disruption and prior tetracycline exposure in an isogenic *E. amylovora* background. Four experimental groups were examined: the WT, an *acrR* mutant (Δ*acrR*), the same mutant after prior tetracycline exposure (Δ*acrR*^Tc^), and a plasmid-complemented Δ*acrR* strain [Δ*acrR*(*acrR*)]. We evaluated tetracycline susceptibility, *acrAB* expression, intracellular tetracycline accumulation, intracellular pH, ATP-associated luminescence, LPS- and cell-envelope-associated phenotypes, virulence-related traits, and responses to exogenous AHL supplementation.

## Materials and Methods

### Bacterial Strains, Mutant Construction, and Culture Conditions

The WT strain used in this study was *E. amylovora* TS3128, which was isolated from pear in the Republic of Korea in 2015 [17]. The Δ*acrR* mutant was constructed in the TS3128 background by λ Red recombination using the one-step PCR-based gene inactivation method [18, 19]. A chloramphenicol resistance cassette amplified from pKD3 with 50-bp homology arms flanking *acrR* was electroporated into TS3128(pKD46) after induction of λ Red recombinase expression with 0.1% L-arabinose. Transformants were selected on Luria-Bertani (LB) agar containing 20 μg/mL chloramphenicol, and replacement of *acrR* was verified by PCR using flanking and cassette-specific primers. The Δ*waaL* mutant used as an LPS-assembly control was constructed in the TS3128 background using the same λ Red recombination procedure. Replacement of *waaL* was verified by PCR using *waaL*-flanking and cassette-specific primers. For complementation, the wild-type *acrR* region was amplified and cloned into pCL1920. The resulting plasmid was introduced into Δ*acrR* by electroporation, and transformants were selected on LB agar containing streptomycin at a final concentration of 100 μg/mL. The presence of the *acrR*-containing plasmid in the transformants was confirmed by PCR, and the resulting complemented strain was designated Δ*acrR*(*acrR*). The strains and their culture conditions are listed in Table S1, whereas the primer sequences used for mutant construction, verification, complementation, and quantitative reverse transcription PCR (qRT-PCR) are listed in Table S2.

### Tetracycline Treatment Conditions

The four experimental groups were cultured under the following conditions. WT, Δ*acrR*, and Δ*acrR*(*acrR*) were cultured without tetracycline, whereas Δ*acrR*^Tc^ was prepared by culturing Δ*acrR* on LB agar containing 10 μg/mL tetracycline at 28°C for 24 h. Thus, Δ*acrR*^Tc^ represented the Δ*acrR* background with a history of tetracycline exposure. The four groups prepared under these conditions were used for MIC and MBC determination and for analyses of cell-envelope ultrastructure, biofilm and extracellular polysaccharide-associated phenotypes, LPS-associated phenotypes and *waaL* expression, pathogenicity, *in planta* *dspE* expression, and AHL supplementation. To examine their responses to tetracycline, all four experimental groups prepared as described above were additionally exposed to 10 μg/mL tetracycline for 6 h before analysis of *acrA* and *acrB* expression, intracellular tetracycline accumulation, intracellular pH, and ATP-associated luminescence.

### MIC and MBC Determination

The minimum inhibitory concentration (MIC) was defined as the lowest tetracycline concentration at which the optical density at 600 nm (OD_600_) did not increase above that of the corresponding uninoculated medium control after incubation. The minimum bactericidal concentration (MBC) was defined as the lowest tetracycline concentration from which no bacterial colonies were recovered following subculture on antibiotic-free Mueller-Hinton agar. Mueller-Hinton broth (MHB; beef extract 2.0 g/L, acid hydrolysate of casein 17.5 g/L, and starch 1.5 g/L; pH 7.3) was used as described previously [20]. Twofold serial dilutions of tetracycline were prepared in MHB to yield final concentrations ranging from 1,024 to 0.25 μg/mL in each well. Bacterial inocula were standardized to 0.5 McFarland (approximately 1.5 × 10^8^ colony-forming units (CFU)/mL) and diluted to yield a final concentration of 1 × 10^5^ CFU/mL in each well. Equal volumes of the antibiotic dilution and bacterial suspension were mixed in 96-well plates and incubated at 28°C for 24 h. For MBC determination, 10 μL aliquots were subcultured onto antibiotic-free Mueller-Hinton agar and incubated at 28°C for 24 h. The MIC and MBC assays were performed using three independent biological replicates, with three technical wells for each group and concentration.

### Quantitative Reverse Transcription PCR

Total RNA was extracted from bacterial cultures and infected immature apple fruit tissues (cv. Fuji) using the PureLink RNA Mini Kit (Invitrogen, USA). For *waaL* expression analysis, Δ*waaL* was included as a negative control. First-strand complementary DNA (cDNA) was synthesized from 10 ng of total RNA using the SuperScript III First-Strand Synthesis Kit (Invitrogen). qRT-PCR was performed in 20 μL reactions containing 2 μL of cDNA, 2× SYBR Green Master Mix (Elpis Biotech Inc., Republic of Korea), and 10 pmol of each primer using a StepOne Real-Time PCR System (Applied Biosystems, USA). Cycling conditions were 95°C for 10 min, followed by 40 cycles of 95°C for 15 sec and 55°C for 1 min, followed by melting-curve analysis. Relative transcript levels of *acrA*, *acrB*, *dspE*, and *waaL* were normalized to *rrs* and calculated using the 2^−ΔΔCt^ method [21]. WT was used as the calibrator for each experiment. For *in planta* expression analysis, immature apple fruits were inoculated as described in the pathogenicity assay. Tissue surrounding the inoculation site was collected at 10 days post-inoculation and used for total RNA extraction using the same procedure described above. All transcript analyses were performed using three independent biological replicates, each analyzed in technical triplicate.

### Quantification of Intracellular Tetracycline

Intracellular tetracycline accumulation was quantified using a competitive enzyme-linked immunosorbent assay (ELISA) kit for tetracyclines (MyBioSource, USA) according to the manufacturer’s instructions. Cells from 10 mL cultures were harvested, washed with phosphate-buffered saline (PBS) to remove extracellular tetracycline, and disrupted by sonication on ice. After centrifugation to remove cell debris, the clarified extracts were subjected to ELISA analysis. Absorbance was measured at 450 nm with reference correction at 630 nm using a SPECTROstar Nano microplate reader (BMG LABTECH, Germany). Intracellular tetracycline concentrations were calculated from the standard curve, corrected for sample dilution, and normalized to the cell pellet mass. The assay was performed using three independent biological replicates, each analyzed in technical triplicate.

### Measurement of Intracellular pH and ATP

Intracellular pH was measured using 2′,7′-bis-(2-carboxyethyl)-5-(and-6)-carboxyfluorescein acetoxymethyl ester (BCECF-AM) (Invitrogen) according to the manufacturer’s instructions. Cells were harvested at mid-log phase, loaded with BCECF-AM, washed three times, and resuspended in measurement buffer. Fluorescence was measured in black microplates using a Synergy H1 microplate reader (Agilent BioTek, USA) at excitation wavelengths of 490 and 440 nm and an emission wavelength of 535 nm. Intracellular pH was calculated from the 490/440 fluorescence ratio using *in situ* calibration with nigericin in high-potassium buffer [22]. Cellular ATP-associated luminescence was measured using the ENLITEN ATP Assay System (Promega, USA) according to the manufacturer’s instructions. Cells were adjusted to OD_600_ = 1.0, and ATP was extracted using a trichloroacetic acid-based procedure. Luminescence was measured using the Synergy H1 microplate reader, and signals were blank-subtracted and normalized to cell biomass. Both assays were performed using three independent biological replicates, each analyzed in technical triplicate.

### Transmission Electron Microscopy

Cell-envelope ultrastructure was examined by transmission electron microscopy. Cells were fixed overnight at 4°C with 2% glutaraldehyde and 2% paraformaldehyde in 50 mM sodium cacodylate buffer (pH 7.4). Samples were then postfixed with 2% osmium tetroxide and 3% potassium hexacyanoferrate for 2 h. The fixed samples were dehydrated through a graded ethanol series, with each dehydration step performed for 20 min. Samples were sequentially infiltrated with mixtures of 100% ethanol and LR White resin at ratios of 2:1, 1:1, and 1:2 for 1.5 h at each step, followed by infiltration with pure LR White resin for 3 h. Samples were transferred to dry capsule molds filled with LR White resin and polymerized at 70°C for 24 h. Ultrathin sections of approximately 80 nm were prepared, mounted on copper grids, and stained with uranyl acetate and lead citrate [23]. Images were acquired at an accelerating voltage of 200 kV using a JEM-2100F transmission electron microscope (JEOL, Tokyo, Japan). Three independently prepared biological samples were examined using identical acquisition settings.

### Biofilm and Extracellular Polysaccharide (EPS) Associated Assays

Biofilm biomass, amylovoran production, levansucrase-associated activity, and cellulose-associated colony phenotypes were examined. Biofilm biomass was quantified by crystal violet staining after cultivation in LB broth at 28°C for 48 h, and the retained stain was measured at 585 nm. Amylovoran production was measured using the cetylpyridinium chloride turbidity assay with culture supernatants obtained after growth in modified basal medium A (MBMA) containing 1% sorbitol for 40 h [24, 25]. Levansucrase-associated activity was measured using culture supernatants incubated with sucrose-containing assay buffer, and absorbance was recorded at 580 nm [26]. Cellulose-associated colony phenotypes were qualitatively assessed based on colony coloration on Congo red-containing medium, with modifications from a previously described method for *E. amylovora* [27]. Briefly, 5 μL of bacterial suspension adjusted to OD_600_ = 0.1 was spotted onto NaCl-free LB agar containing 40 μg/mL Congo red and incubated at 28°C for 48 h. All assays were performed using three independent biological replicates.

### LPS Extraction and Sodium Dodecyl Sulfate–Polyacrylamide Gel Electrophoresis (SDS-PAGE) Analysis

LPS was extracted using an LPS extraction kit (Sigma-Aldrich, USA) according to the manufacturer’s instructions. Δ*waaL* was included as a negative control. Bacterial cultures were adjusted to the same OD_600_, and equal volumes were harvested for LPS extraction. Bacterial pellets were suspended in two pellet volumes of lysis buffer and disrupted on ice for a total of 2 min using 10-sec pulses with a Q125 sonicator (Qsonica, USA) operated at 10 W. The lysates were treated with proteinase K at a final concentration of 0.1 mg/mL for 60 min at 60°C. Samples were mixed with an equal volume of 2× Laemmli sample buffer, heated at 95°C for 3 min, and separated by SDS-PAGE using a 5% stacking gel and a 12% resolving gel at 140 V for 55 min [28]. Following electrophoresis, LPS-associated banding patterns were visualized by Brilliant Blue G staining. The intensity of the major LPS-associated band was quantified using GelAnalyzer version 23.1.1 and normalized to the WT value, which was set to 1. The experiment was performed using three independent biological replicates.

### Pathogenicity Tests

Pathogenicity was evaluated using immature apple fruits as described previously, with minor modifications [29]. Bacterial suspensions were adjusted to OD_600_ = 0.1 (approximately 3.1 × 10^8^ CFU/mL) in 10 mM MgCl_2_. Surface-sterilized immature apple fruits were wounded with a sterile No. 1 cork borer and inoculated with 50 μL of bacterial suspension. Fruits were incubated at 28°C for 10 days. Disease symptoms were photographed at 10 days post-inoculation. For bacterial population analysis, tissue samples centered on the inoculation site were collected using a sterile No. 1 cork borer at 10 days post-inoculation and macerated in 1 mL of 10 mM MgCl_2_. The resulting tissue homogenates were serially diluted and plated on LB agar, and bacterial populations were calculated as CFU/mL of tissue homogenate based on the colony count, dilution factor, and plated volume. The experiment was performed using three independent biological replicates, each including five fruit samples per group.

### AHL Supplementation Assay in Immature Apple Fruits

The assay was performed as described above for the pathogenicity assay, except that bacterial suspensions were individually supplemented with *N*-(3-oxohexanoyl)-_L_-homoserine lactone (OHHL; Santa Cruz Biotechnology, USA; sc-205399A), *N*-hexanoyl-_L_-homoserine lactone (HHL; Sigma-Aldrich, USA; 56395), or *N*-(3-oxooctanoyl)-_L_-homoserine lactone (OOHL; Santa Cruz Biotechnology, USA; sc-205400A) at 10 μM. AHL stocks were prepared in dimethyl sulfoxide (DMSO), and all inocula, including the no-AHL control, contained 1% (v/v) DMSO. A 50 μL aliquot of each bacterial suspension was used for inoculation. OHHL and HHL were used as short-chain AHL treatments, whereas OOHL was included as an additional AHL comparison treatment. Disease severity was scored at 10 days post-inoculation based on the proportion of the fruit surface exhibiting visible necrotic symptoms using a 0–5 ordinal scale: 0, no visible necrosis; 1, > 0–20% of the fruit surface affected; 2, > 20–40%; 3, > 40–60%; 4, > 60–80%; and 5, > 80–100%. The assay included three independent biological replicates with five fruit samples per treatment.

### Statistical Analyses

Data are presented as mean ± standard deviation (SD). Technical replicates were averaged before statistical analysis. qRT-PCR data were analyzed using ΔCt values, whereas 2^−ΔΔCt^ values were used for graphical presentation. Comparisons among experimental groups were performed using one-way analysis of variance (ANOVA) followed by Tukey’s multiple-comparison test. For analyses of LPS-associated band intensity and *waaL* expression, Δ*waaL* was included as a negative control but was excluded from quantitative statistical comparisons because no detectable LPS-associated band or *waaL* transcript signal was obtained. CFU data were log_10_-transformed before analysis. Disease severity scores were analyzed separately within each supplementation condition using the Kruskal–Wallis test followed by Dunn’s multiple-comparison test. Differences were considered significant at *P* < 0.05.

## Results

### Tetracycline MIC and MBC

To determine whether *acrR* disruption and prior tetracycline exposure affected tetracycline susceptibility, MIC and MBC values were determined for WT, Δ*acrR*, Δ*acrR*^Tc^, and Δ*acrR*(*acrR*) using MIC and MBC assays (Table 1). The tetracycline MIC and MBC of WT were 1 and 16 μg/mL, respectively. For Δ*acrR*, the MIC increased to 4 μg/mL and the MBC increased to 64 μg/mL. The prior tetracycline-exposed Δ*acrR* group (Δ*acrR*^Tc^) showed the highest values, with an MIC of 16 μg/mL and an MBC of 512 μg/mL. The complemented strain Δ*acrR*(*acrR*) exhibited an MIC of 4 μg/mL and an MBC of 64 μg/mL. Thus, the MIC values of Δ*acrR*, Δ*acrR*^Tc^, and Δ*acrR*(*acrR*) were 4-, 16-, and 4-fold higher than that of WT, respectively, whereas their MBC values were 4-, 32-, and 4-fold higher than that of WT.

### *acrA* and *acrB* Expression

To examine whether differences in tetracycline susceptibility were associated with changes in *acrAB* expression, relative transcript levels of *acrA* and *acrB* were measured by qRT-PCR in WT, Δ*acrR*, Δ*acrR*^Tc^, and Δ*acrR*(*acrR*) (Fig. 1). The *acrA* transcript level in Δ*acrR*^Tc^ was approximately sevenfold higher than that in WT and was significantly higher than those in the other groups (Fig. 1A). By comparison, Δ*acrR* did not differ significantly from WT or Δ*acrR*(*acrR*). Similarly, the *acrB* transcript level in Δ*acrR*^Tc^ was approximately 14-fold higher than that in WT and was significantly higher than those in the other groups (Fig. 1B). No significant differences in *acrB* expression were detected among WT, Δ*acrR*, and Δ*acrR*(*acrR*).

### Intracellular Tetracycline, pH, and ATP-Associated Luminescence

To determine whether changes in *acrAB* expression were accompanied by changes in intracellular tetracycline accumulation and cellular physiology, intracellular tetracycline accumulation, intracellular pH, and ATP-associated luminescence were compared among the four experimental groups (Fig. 2). Intracellular tetracycline concentrations were approximately 1.9 ng/g cell mass in WT, 1.7 ng/g cell mass in Δ*acrR*, and 1.9 ng/g cell mass in Δ*acrR*(*acrR*) (Fig. 2A). In contrast, Δ*acrR*^Tc^ contained approximately 1.1 ng/g cell mass, which was significantly lower than those in the other groups. The intracellular pH values of WT, Δ*acrR*, and Δ*acrR*(*acrR*) were approximately 6.2, 6.25, and 6.3, respectively (Fig. 2B). Δ*acrR*^Tc^ showed a significantly lower intracellular pH of approximately 5.6. ATP-associated luminescence was approximately 1.7 × 10^4^ relative luminescence units (RLU) in WT and 1.9 × 10^4^ RLU in Δ*acrR* and Δ*acrR*(*acrR*) (Fig. 2C). By comparison, Δ*acrR*^Tc^ showed a significantly lower signal of approximately 6.0 × 10³ RLU.

### Cell-Envelope Ultrastructure

To determine whether prior tetracycline exposure was associated with changes in cell-envelope morphology, cell-envelope ultrastructure was examined by transmission electron microscopy in WT, Δ*acrR*, Δ*acrR*^Tc^, and Δ*acrR*(*acrR*) (Fig. 3). WT, Δ*acrR*, and Δ*acrR*(*acrR*) displayed relatively uniform cell-envelope structures (Fig. 3A, B, and D). In contrast, Δ*acrR*^Tc^ exhibited an irregular electron-dense layer along the cell surface (Fig. 3C). A comparable surface-layer phenotype was not evident in WT, Δ*acrR*, or Δ*acrR*(*acrR*).

### Biofilm and EPS-Associated Phenotypes

To examine whether the cell-envelope phenotype was accompanied by changes in surface-associated traits, biofilm biomass, amylovoran production, levansucrase-associated activity, and Congo red-associated colony coloration were examined in WT, Δ*acrR*, Δ*acrR*^Tc^, and Δ*acrR*(*acrR*) (Fig. 4). Biofilm biomass was significantly lower in Δ*acrR*^Tc^ than in WT, Δ*acrR*, and Δ*acrR*(*acrR*) (Fig. 4A). In contrast, no significant differences were detected among the tested groups in amylovoran production or levansucrase-associated activity (Fig. 4B and 4C). On Congo red-containing medium, Δ*acrR*^Tc^ exhibited visibly weaker colony coloration than WT, Δ*acrR*, and Δ*acrR*(*acrR*) (Fig. 4D).

### LPS-Associated Band Intensity and *waaL* Expression

To assess whether the altered cell-envelope phenotype was associated with LPS-related changes, LPS preparations from WT, Δ*acrR*, Δ*acrR*^Tc^, Δ*acrR*(*acrR*), and Δ*waaL* were analyzed by SDS-PAGE (Fig. 5A). Δ*acrR*^Tc^ showed a more intense major LPS-associated band than WT, Δ*acrR*, and Δ*acrR*(*acrR*), whereas the corresponding band was not detected in Δ*waaL*. Densitometric analysis showed that the relative band intensity in Δ*acrR*^Tc^ was approximately 1.5-fold that of WT and was significantly higher than the corresponding values in WT, Δ*acrR*, and Δ*acrR*(*acrR*) (Fig. 5B). Relative *waaL* transcript levels were measured by qRT-PCR (Fig. 5C). The *waaL* transcript level in Δ*acrR*^Tc^ was approximately 1.6-fold that of WT and was significantly higher than levels in WT, Δ*acrR*, and Δ*acrR*(*acrR*).

### Virulence and *dspE* Expression in Immature Apple Fruits

To determine whether the physiological and cell-envelope changes were accompanied by altered virulence, pathogenicity of WT, Δ*acrR*, Δ*acrR*^Tc^, and Δ*acrR*(*acrR*) was evaluated in immature apple fruits (Fig. 6). At 10 days post-inoculation, WT, Δ*acrR*, and Δ*acrR*(*acrR*) produced extensive necrotic symptoms, whereas Δ*acrR*^Tc^ showed visibly reduced necrotic symptom development (Fig. 6A). Bacterial populations in tissue homogenates from fruits inoculated with Δ*acrR*^Tc^ were approximately 5.1 log_10_ CFU/mL, significantly lower than those recovered from the other groups, which ranged from approximately 8.4 to 8.7 log_10_ CFU/mL (Fig. 6B). Relative *dspE* transcript levels were also measured in infected fruit tissues (Fig. 6C). The *dspE* transcript level in Δ*acrR*^Tc^ was approximately 0.14-fold that of WT and was significantly lower than those in the other groups. The transcript levels in Δ*acrR* and Δ*acrR*(*acrR*) were approximately 0.67- and 0.84-fold relative to WT, respectively, and did not differ significantly from the WT level.

### Virulence under AHL Supplementation

To examine whether the virulence phenotypes differed in response to exogenous AHL supplementation, disease severity was evaluated in immature apple fruits inoculated with WT, Δ*acrR*, Δ*acrR*^Tc^, and Δ*acrR*(*acrR*) under the DMSO control condition or following supplementation with OHHL, HHL, or OOHL at 10 μM (Fig. 7). In the DMSO control, Δ*acrR*^Tc^ showed a significantly lower disease severity score than WT, Δ*acrR*, and Δ*acrR*(*acrR*) (Fig. 7A). A distinct group-dependent pattern was observed under OHHL supplementation. WT and Δ*acrR*(*acrR*) showed the highest disease severity scores, whereas Δ*acrR* showed an intermediate score and Δ*acrR*^Tc^ remained at the lowest level (Fig. 7B). In contrast, under HHL supplementation, Δ*acrR* did not differ significantly from WT or Δ*acrR*(*acrR*), whereas Δ*acrR*^Tc^ remained significantly lower than the other groups (Fig. 7C). Similarly, under OOHL supplementation, no significant differences were observed among WT, Δ*acrR*, and Δ*acrR*(*acrR*), whereas Δ*acrR*^Tc^ again showed the lowest disease severity score (Fig. 7D). Thus, among the AHLs tested, a reduced disease severity score for Δ*acrR* relative to WT and Δ*acrR*(*acrR*) was specifically observed under OHHL supplementation, whereas Δ*acrR*^Tc^ maintained a reduced disease severity phenotype under all AHL conditions.

## Discussion

This study compared the phenotype of the *acrR* mutant of *E. amylovora* with that of the same mutant after prior tetracycline exposure. The Δ*acrR* mutant showed higher tetracycline MIC and MBC values than WT but did not show marked *acrA* or *acrB* overexpression or reduced virulence. In contrast, the prior tetracycline-exposed Δ*acrR* group (Δ*acrR*^Tc^) showed the highest MIC and MBC values and was additionally characterized by increased *acrAB* expression, reduced intracellular tetracycline accumulation, lower intracellular pH, reduced ATP-associated luminescence, altered cell-envelope and surface-associated phenotypes, and reduced virulence. Thus, within the experimental groups tested, the prior tetracycline-exposed Δ*acrR* group exhibited additional physiological, cell-envelope, and virulence-associated changes that were not observed in the unexposed Δ*acrR* mutant.

AcrR is a local transcriptional repressor of the *acrAB* operon, and impairment of this regulatory system has been associated with reduced tetracycline susceptibility in several Gram-negative bacteria [8, 10, 11]. The fourfold increases in MIC and MBC observed in Δ*acrR* are consistent with a reduction in tetracycline susceptibility. These findings are consistent with tetracycline serving as a substrate of the AcrAB efflux system and suggest that AcrR-dependent regulation contributes to tetracycline susceptibility through control of AcrAB.

Previous studies in *E. coli* have shown that tetracycline exposure can enhance AcrAB–TolC-dependent efflux responses and increase *acrAB*-*tolC* expression during continued antibiotic exposure [12, 13]. In the present study, Δ*acrR*^Tc^ showed markedly increased *acrA* and *acrB* expression together with significantly reduced intracellular tetracycline accumulation compared with the other groups. The coordinated increase in *acrAB* expression and decrease in intracellular tetracycline are consistent with enhanced AcrAB-mediated tetracycline export and support the interpretation that tetracycline functions as a substrate of the AcrAB efflux system in *E. amylovora*. These findings further suggest that disruption of AcrR-mediated regulation, together with prior tetracycline exposure, promotes a state in which AcrAB-dependent tetracycline transport is enhanced.

Complementation of *acrR* did not restore the tetracycline MIC and MBC to WT levels, suggesting that the susceptibility phenotype was not fully reversible under the complementation conditions used. Nevertheless, the markedly increased *acrAB* expression and reduced intracellular tetracycline accumulation in Δ*acrR*^Tc^ support the involvement of the AcrR–AcrAB regulatory system in tetracycline transport.

Because AcrB couples drug export to proton translocation [7, 30], we examined whether the enhanced *acrAB* expression in Δ*acrR*^Tc^ was accompanied by changes in proton and energy homeostasis. The proton motive force also contributes to ATP synthesis in bacteria [31], suggesting that changes in proton homeostasis may influence cellular ATP production. Δ*acrR*^Tc^ showed both a lower intracellular pH and reduced ATP-associated luminescence compared with the other groups. Together, these findings indicate that enhanced *acrAB* expression in Δ*acrR*^Tc^ was accompanied by alterations in proton and energy homeostasis.

Cell-envelope analysis revealed an irregular electron-dense surface layer specifically in Δ*acrR*^Tc^, whereas comparable changes were not observed in WT, Δ*acrR*, or Δ*acrR*(*acrR*). This phenotype was not accompanied by a general increase in extracellular matrix-associated traits, as Δ*acrR*^Tc^ showed reduced biofilm formation and weaker cellulose-associated colony coloration, while amylovoran production and levansucrase-associated activity remained unchanged. In contrast, Δ*acrR*^Tc^ showed increased *waaL* expression and greater intensity of a major LPS-associated band. WaaL catalyzes the ligation of O-antigen to the LPS core, and the absence of the corresponding band in Δ*waaL* supports its association with WaaL-dependent LPS assembly [32]. Notably, these cell-envelope- and LPS-associated changes were not evident in Δ*acrR* but were observed in Δ*acrR*^Tc^, indicating that prior tetracycline exposure was associated with additional envelope-related changes in the same *acrR*-deficient background. Together, these findings suggest that the altered cell-envelope phenotype in Δ*acrR*^Tc^ was more closely associated with LPS-related changes than with increased extracellular polysaccharide production.

In addition to these physiological and cell-envelope changes, virulence analysis showed that Δ*acrR*^Tc^ produced weaker necrotic symptoms, lower bacterial populations, and reduced *in planta* *dspE* expression compared with WT, Δ*acrR*, and Δ*acrR*(*acrR*). The type III secretion system and its effector DspE are central to the pathogenicity of *E. amylovora* [33, 34]. In other Gram-negative bacterial systems, type III protein export depends on the proton motive force and the activity of a secretion-associated ATPase [35, 36]. Notably, the reduced virulence of Δ*acrR*^Tc^ occurred together with markedly increased *acrAB* expression, reduced intracellular tetracycline accumulation, lower intracellular pH, reduced ATP-associated luminescence, and cell-envelope-associated changes. These coordinated physiological alterations indicate that the enhanced AcrAB-associated tetracycline response in Δ*acrR*^Tc^ was accompanied by a broader change in cellular physiology. The concurrent reduction in *dspE* expression, bacterial population, and necrotic symptom development further suggests that this altered physiological state was associated with impaired virulence-related functions, including the type III secretion system, ultimately resulting in reduced pathogenicity of Δ*acrR*^Tc^.

Because *E. amylovora* produces AHL signals [37, 38] and RND-type efflux systems have been associated with AHL transport and quorum-sensing regulation in other Gram-negative bacteria [39], we next examined whether the AcrR–AcrAB regulatory state might also be related to virulence-associated signaling. Among the AHLs tested, OHHL produced a distinct group-dependent virulence pattern. Δ*acrR* showed lower disease severity than WT and Δ*acrR*(*acrR*) under OHHL supplementation, whereas this difference was not observed under HHL or OOHL supplementation. This OHHL-specific phenotype suggests a selective relationship between AcrR–AcrAB regulation and OHHL-associated signaling and raises the possibility that OHHL may represent an AcrAB-associated substrate involved in virulence regulation. In contrast, Δ*acrR*^Tc^ maintained low disease severity under all AHL supplementation conditions. Because Δ*acrR*^Tc^ was characterized by markedly increased *acrAB* expression together with altered intracellular tetracycline accumulation, pH, ATP-associated luminescence, and cell-envelope phenotypes, its persistently reduced virulence suggests that the broader physiological state associated with enhanced AcrAB activity was not restored by exogenous AHL supplementation. Collectively, these findings link the AcrR–AcrAB regulatory system not only to tetracycline transport but also to OHHL-associated virulence signaling in *E. amylovora*.

Overall, these findings support an important role for the AcrR–AcrAB regulatory system in the response of *E. amylovora* to tetracycline and are consistent with tetracycline serving as a substrate of the AcrAB efflux system. The enhanced tetracycline resistance and AcrAB response observed in Δ*acrR*^Tc^ further suggest that prior tetracycline exposure can strengthen the subsequent efflux-associated response in an *acrR*-deficient background. The accompanying physiological, cell-envelope, and virulence-associated changes indicate that this response extends beyond antibiotic susceptibility and is linked to broader changes in bacterial physiology and pathogenicity. In addition, the OHHL-specific phenotype raises the possibility that the AcrR–AcrAB system is also associated with specific AHL-dependent virulence signaling, potentially involving OHHL as an AcrAB-associated substrate. Together, these findings connect AcrR–AcrAB regulation with tetracycline transport, physiological adaptation, and virulence-associated signaling in *E. amylovora*.

## Supplemental Materials

Supplementary data for this paper are available on-line only at http://jmb.or.kr.



## Figures and Tables

**Fig. 1:**
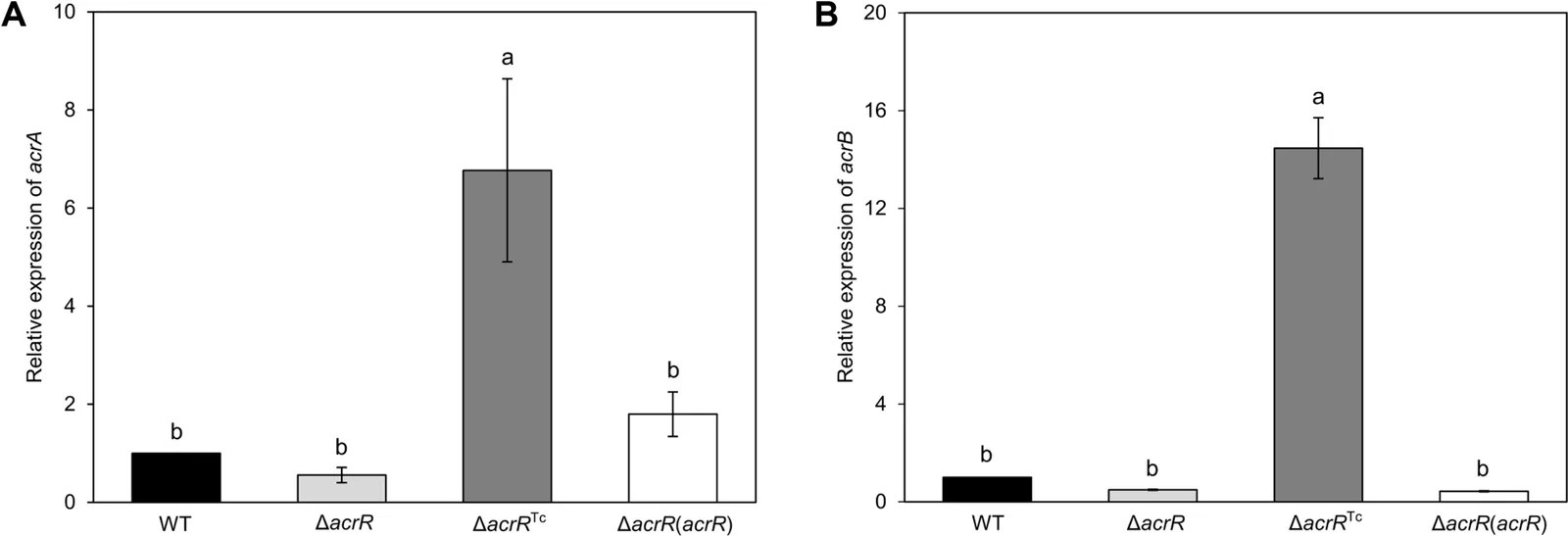
Relative expression of *acrA* and *acrB*. (**A**) Relative transcript level of *acrA*. (**B**) Relative transcript level of *acrB*. Before RNA extraction, all four experimental groups were subjected to the same tetracycline challenge (10 μg/mL) for 6 h. Transcript levels were determined by quantitative reverse transcription PCR (qRT-PCR), normalized to *rrs* (16S rRNA), and expressed relative to the WT value, which was set to 1. Bars represent the mean ± SD of three independent biological replicates, each analyzed in technical triplicate. Different lowercase letters indicate significant differences among groups according to one-way ANOVA followed by Tukey’s multiple-comparison test (*P* < 0.05). Δ*acrR*^Tc^ denotes the Δ*acrR* mutant after prior tetracycline exposure, and Δ*acrR*(*acrR*) denotes the complemented mutant.

**Fig. 2:**
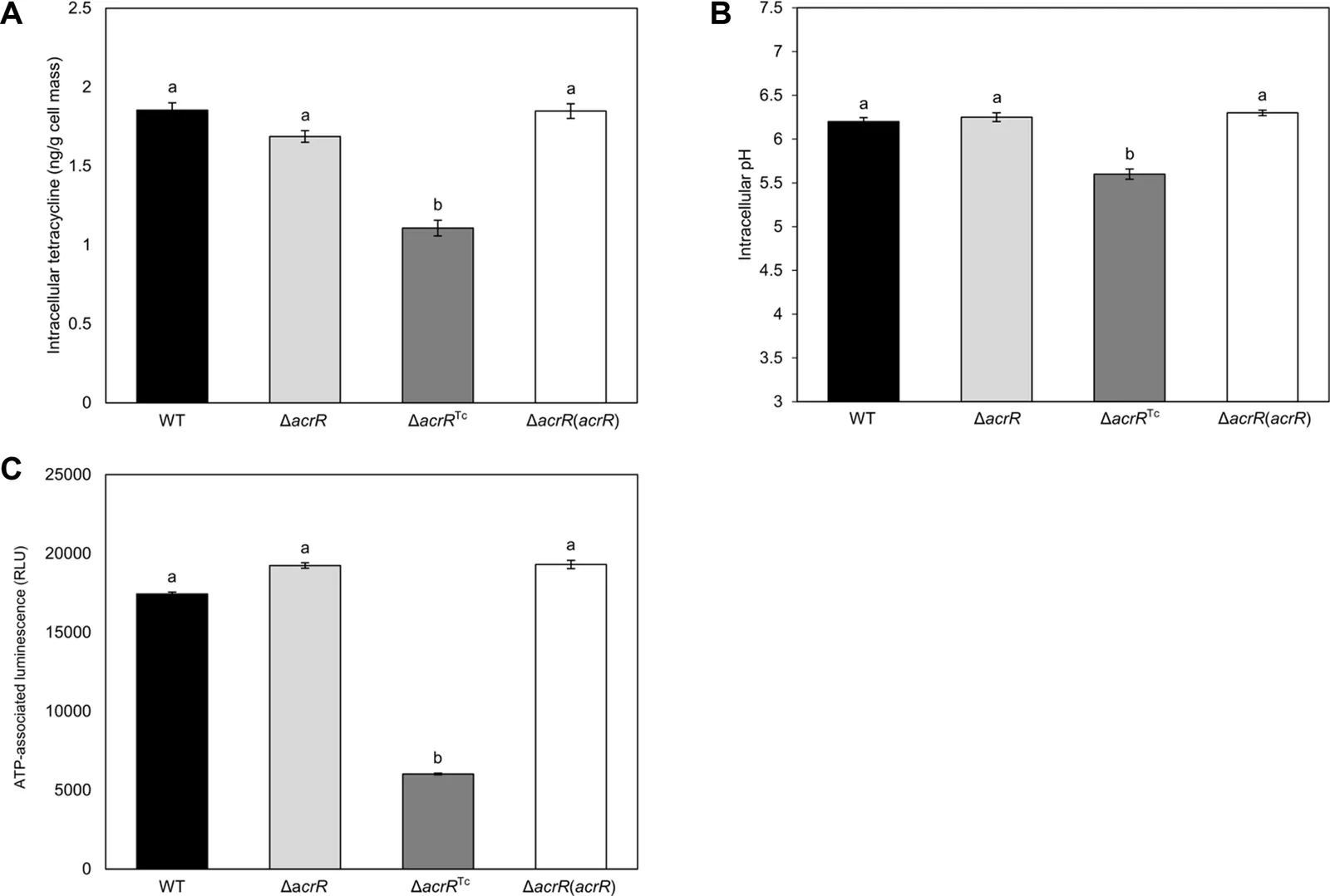
Intracellular tetracycline, pH, and ATP-associated luminescence. (**A**) Intracellular tetracycline concentration normalized to cell pellet mass. (**B**) Intracellular pH. (**C**) ATP-associated luminescence expressed as relative luminescence units (RLU). Before analysis, all four experimental groups were subjected to the same tetracycline challenge (10 μg/mL) for 6 h. Bars represent the mean ± SD of three independent biological replicates, each analyzed in technical triplicate. Different lowercase letters indicate significant differences among groups according to one-way ANOVA followed by Tukey’s multiple-comparison test (*P* < 0.05).

**Fig. 3:**
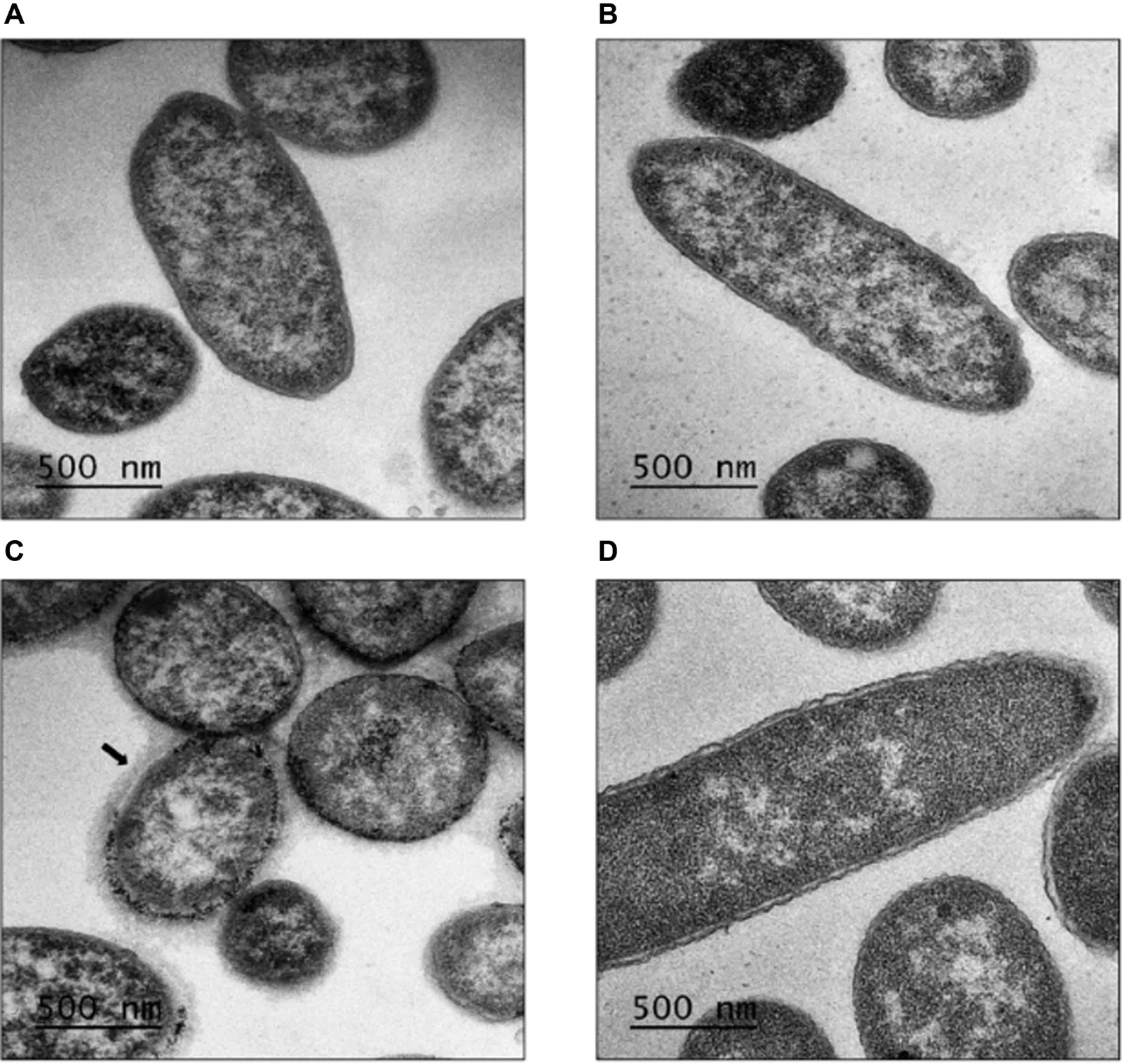
Cell-envelope ultrastructure of the experimental groups. (**A**) WT. (**B**) Δ*acrR*. (**C**) Δ*acrR*^Tc^. (**D**) Δ*acrR*(*acrR*). Representative transmission electron micrographs are shown; similar observations were obtained in three independent experiments. Black arrows in panel C indicate the thickened electron-dense surface layer. All images were acquired under the same conditions. Scale bars, 500 nm.

**Fig. 4:**
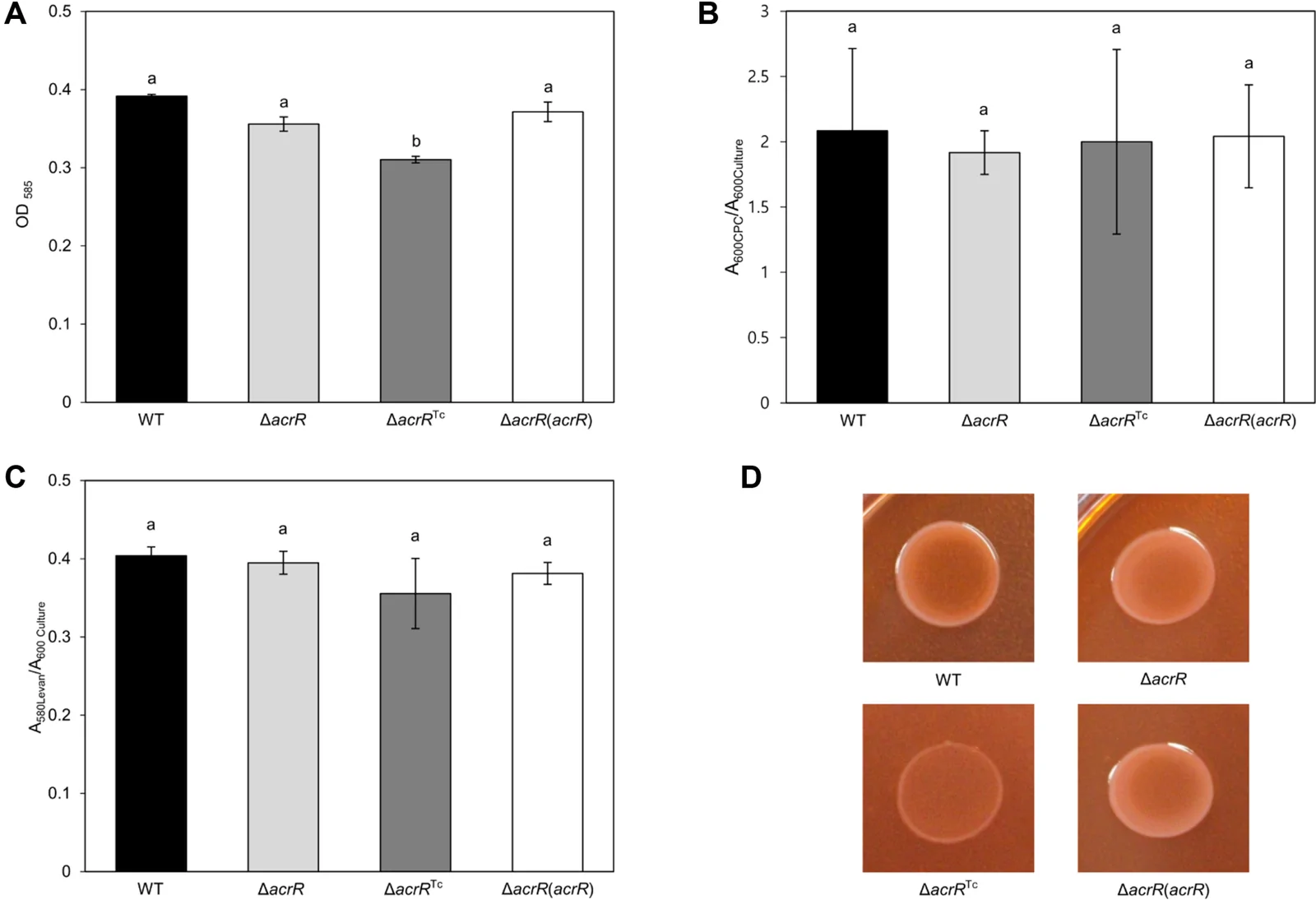
Biofilm and EPS-associated phenotypes. (**A**) Biofilm biomass measured at OD_585_. (**B**) Amylovoran production expressed as the ratio of absorbance after cetylpyridinium chloride (CPC) treatment to culture turbidity, calculated as A_600CPC_/A_600culture_. (**C**) Levansucrase-associated activity expressed as A_580_(levan)/A_600_(culture). (**D**) Representative colony coloration on Congo red-containing medium. Bars in (**A–C**) represent the mean ± SD of three independent biological replicates. Different lowercase letters indicate significant differences among groups according to one-way ANOVA followed by Tukey’s multiple-comparison test (*P* < 0.05).

**Fig. 5:**
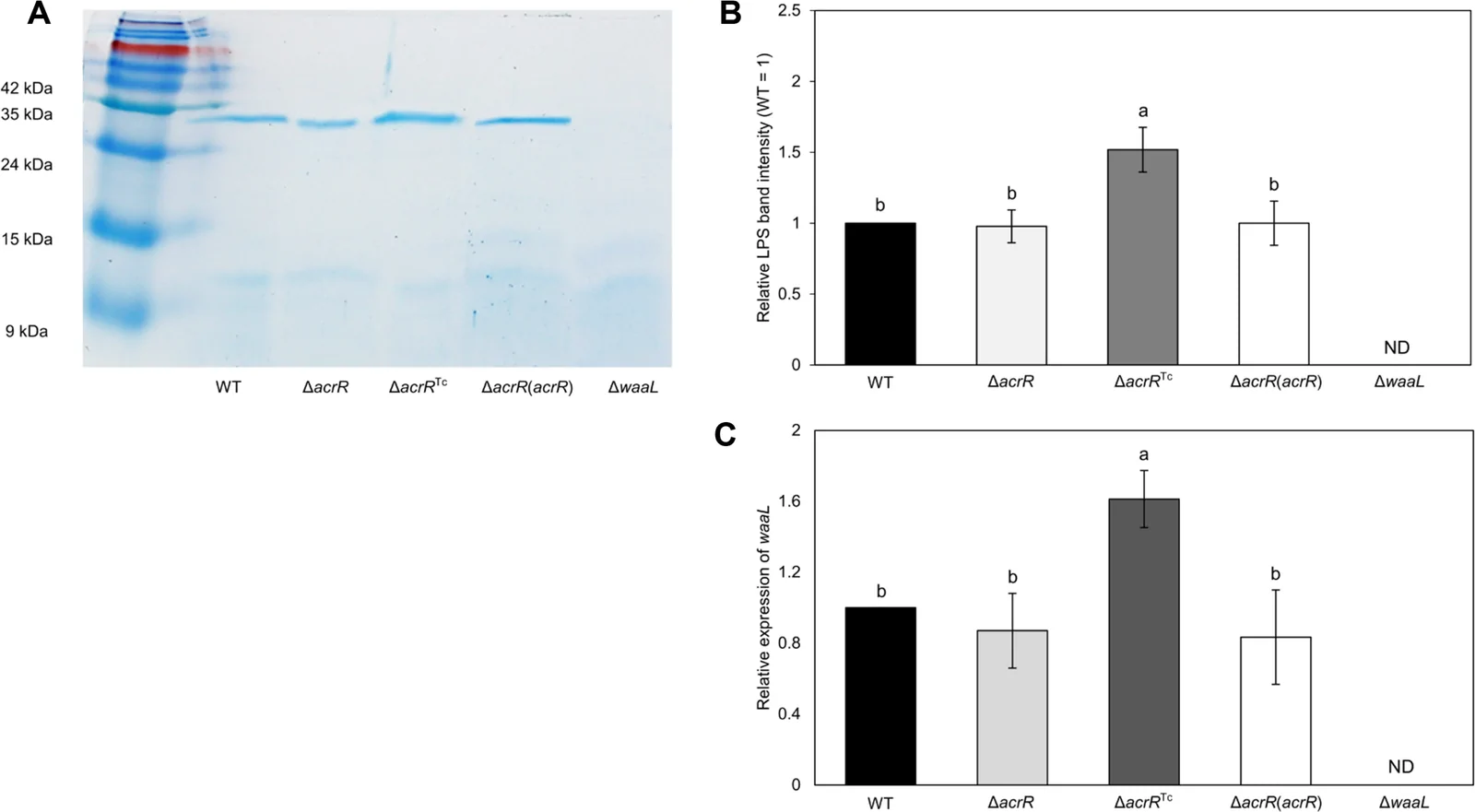
LPS profiles and *waaL* expression. (**A**) SDS-PAGE profiles of LPS preparations from WT, Δ*acrR*, Δ*acrR*^Tc^, Δ*acrR*(*acrR*), and Δ*waaL*. Molecular mass markers are indicated in kilodaltons. (**B**) Densitometric quantification of the major LPS-associated band normalized to the WT value, which was set to 1. (**C**) Relative *waaL* transcript levels determined by qRT-PCR and normalized to *rrs* (16S rRNA). The gel is representative of three independent biological replicates. Bars represent the mean ± SD of three independent biological replicates; qRT-PCR measurements in (**C**) were performed in technical triplicate. Different lowercase letters indicate significant differences among groups according to one-way ANOVA followed by Tukey’s multiple-comparison test (*P* < 0.05). ND, not detected.

**Fig. 6:**
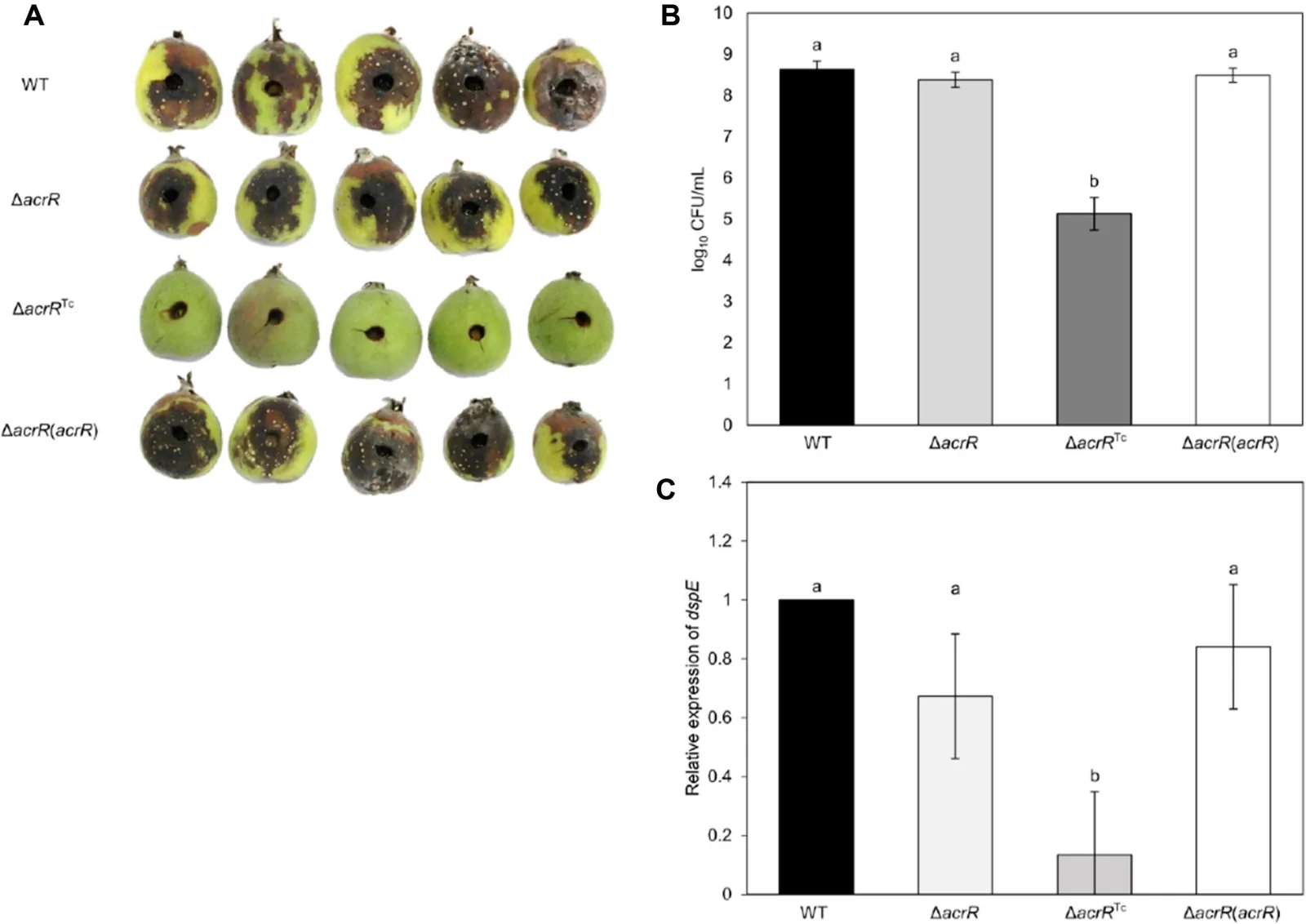
Virulence and *dspE* expression in immature apple fruits. (**A**) Representative symptoms at 10 days post-inoculation. (**B**) Bacterial populations in tissue homogenates at 10 days post-inoculation, expressed as log_10_ CFU/mL. (**C**) Relative *dspE* transcript levels in inoculated fruit tissues determined by qRT-PCR, normalized to *rrs* (16S rRNA), and expressed relative to the WT value, which was set to 1. Images in (**A**) are representative of three independent biological replicates. Data in (**B**) and (**C**) represent the mean ± SD of three independent biological replicates; each replicate included five fruit samples per group, and qRT-PCR measurements in (**C**) were performed in technical triplicate. Different lowercase letters indicate significant differences among groups according to one-way ANOVA followed by Tukey’s multiple-comparison test (*P* < 0.05). Bacterial population data were log_10_-transformed before statistical analysis.

**Fig. 7:**
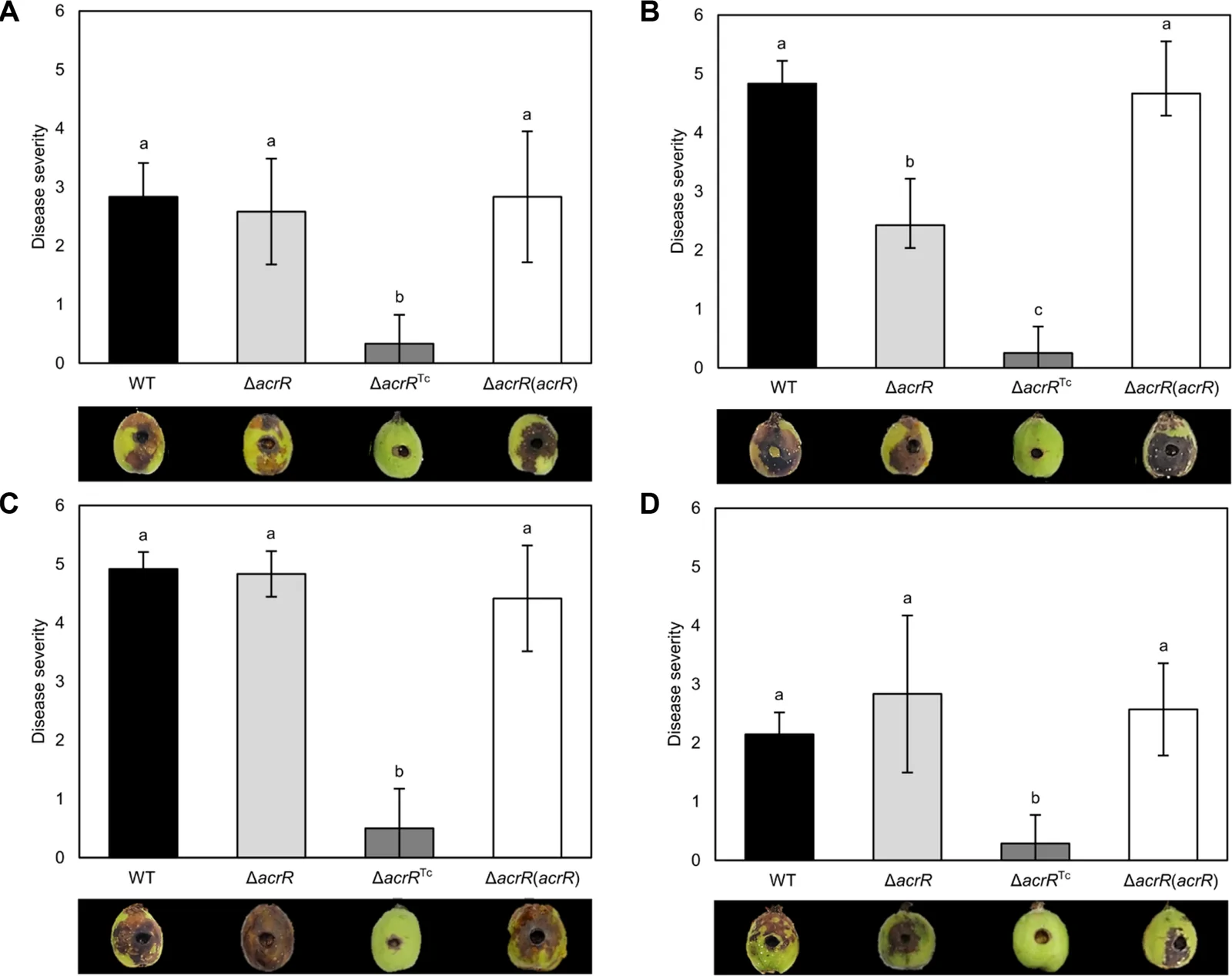
Virulence under AHL supplementation. (**A**) 1% (v/v) DMSO control. (**B**) *N*-(3-oxohexanoyl)-_L_-homoserine lactone (OHHL). (**C**) *N*-hexanoyl-_L_-homoserine lactone (HHL). (**D**) *N*-(3-oxooctanoyl)-_L_-homoserine lactone (OOHL). Each AHL was added at a final concentration of 10 μM, and disease severity was scored at 10 days post-inoculation on a 0–5 ordinal scale based on the proportion of the fruit surface exhibiting visible necrotic symptoms, as described in Materials and Methods. Representative fruit images are shown below the corresponding graphs. Bars represent the mean ± SD of three independent biological replicates, each including five fruit samples per group treatment combination. Different lowercase letters indicate significant differences among groups within each supplementation condition according to the Kruskal–Wallis test followed by Dunn’s multiple-comparison test (*P* < 0.05).

**Table 1:** MIC and MBC values for tetracycline in WT and *acrR*-related groups.

Antibiotic	WT	Δ*acrR*	Δ*acrR*^Tc^	Δ*acrR*(*acrR*)
Tetracycline	MIC (μg/mL)			
	1	4	16	4
	MBC (μg/mL)			
	16	64	512	64

Notes: MIC, minimum inhibitory concentration; MBC, minimum bactericidal concentration. Values are expressed as μg/mL. WT, wild type; Δ*acrR*, *acrR* mutant; Δ*acrR*^Tc^, tetracycline-exposed Δ*acrR* group; Δ*acrR*(*acrR*), complemented Δ*acrR* strain.
